# Detection of early cartilage damage: feasibility and potential of gagCEST imaging at 7T

**DOI:** 10.1007/s00330-017-5277-y

**Published:** 2018-01-30

**Authors:** Sander Brinkhof, Razmara Nizak, Vitaliy Khlebnikov, Jeanine J. Prompers, Dennis W.J. Klomp, Daniel B.F. Saris

**Affiliations:** 1 Department of Radiology, UMC Utrecht, Heidelberglaan 100, 3584 CX Utrecht, The Netherlands; 2 Department of Orthopaedics, UMC Utrecht, Utrecht, The Netherlands; 3 MIRA institute for Biomedical Technology and Technical Medicine, University of Twente, Enschede, The Netherlands; 4 Department of Orthopaedics, Mayo Clinic, Rochester, Minnesota USA

**Keywords:** Cartilage, Glycosaminoglycans, Knee, Magnetic resonance imaging, Osteoarthritis

## Abstract

**Objectives:**

The purpose was to implement a fast 3D glycosaminoglycan Chemical Exchange Saturation Transfer (gagCEST) sequence at 7 T, test stability and reproducibility in cartilage in the knee in healthy volunteers, and evaluate clinical applicability in cartilage repair patients.

**Methods:**

Experiments were carried out on a 7-T scanner using a volume transmit coil and a 32-channel receiver wrap-around knee coil. The 3D gagCEST measurement had an acquisition time of 7 min. Signal stability and reproducibility of the GAG effect were assessed in eight healthy volunteers. Clinical applicability of the method was demonstrated in five patients before cartilage repair surgery.

**Results:**

Coefficient of variation of the gagCEST signal was 1.9%. The reproducibility of the GAG effect measurements was good in the medial condyle (ICC = 0.87) and excellent in the lateral condyle (ICC = 0.97). GAG effect measurements in healthy cartilage ranged from 2.6%-12.4% compared with 1.3%-5.1% in damaged cartilage. Difference in GAG measurement between healthy cartilage and damaged cartilage was significant (*p* < 0.05).

**Conclusions:**

A fast 3D gagCEST sequence was applied at 7 T for use in cartilage in the knee, acquired within a clinically feasible scan time of 7 min. We demonstrated that the method has high stability, reproducibility and clinical applicability.

**Key Points:**

*• gagCEST measurements are stable and reproducible*

*• A non-invasive GAG measurement with gagCEST can be acquired in 7 min*

*• gagCEST is able to discriminate between healthy and damaged cartilage*

**Electronic supplementary material:**

The online version of this article (10.1007/s00330-017-5277-y) contains supplementary material, which is available to authorized users.

## Introduction

With the ageing of our society, the prevalence of degenerative diseases, such as osteoarthritis (OA), has increased [1]. OA is a degenerative whole-joint disease that affects the articular cartilage. Since cartilage tissue has a limited ability to regenerate, early identification of cartilage damage improves chances of successful treatment and prognosis [2, 3]. Early-stage OA and early-stage cartilage damage in general are subject to delicate changes in biochemical composition, i.e. a loss of glycosaminoglycans (GAG), on the surface of the cartilage [4]). The measurement of reductions in GAG is a promising approach for the diagnosis and treatment monitoring of early stage OA. MRI is an excellent modality to visualise cartilage morphology; however, a standard anatomical MRI is not sufficient to visualise early stage OA.

Quantitative MRI has been used increasingly over the past years to quantify GAG content in vivo in OA [5, 6]. Initial studies focused on the application of delayed gadolinium enhanced MRI of cartilage (dGEMRIC) [7, 8] to measure the GAG content indirectly. This technique is based on the distribution of negatively charged ions of a gadolinium-based contrast agent in cartilage, which is inversely proportional to the GAG content [9]. Another technique to assess GAG content, without the use of a contrast agent, is T1ρ mapping. In this technique, spinlock pulses of different durations are applied to assess the T1ρ relaxation time, which is lower in water associated with large macromolecules, such as GAG, as compared to free water. However, it is still disputed whether T1ρ is directly correlated with the GAG content [10]. Alternatively, sodium (^23^Na) MRI measures the sodium ions in the interstitial fluid in the cartilage [11–13]. Sodium counterbalances the negative charge of the sulphate and carboxyl groups of GAG. A lower fixed charge density (FCD), and thus a loss of GAG, causes a loss of sodium ions from cartilage [13]. However, for ^23^Na MRI dedicated MRI coils are required, which are highly experimental and not widely available.

In contrast to these assessments of GAG, chemical exchange saturation transfer (CEST) directly quantifies the GAG content based on the chemical exchange of its labile hydroxyl (-OH) protons with the bulk water [14–16]. These exchangeable protons resonate at a different frequency compared with bulk water protons and are saturated via selective radiofrequency (RF) irradiation. Because of the exchange, the saturation is transferred to the bulk water pool, which ultimately results in large contrast enhancement factors [14, 17–19]. The quantification of GAG in articular cartilage with the use of CEST, i.e. gagCEST, has a high potential for the examination of cartilage degeneration and hence diagnosis of early stage OA. However, in previous applications gagCEST data were mostly acquired in 2D because 3D sequences are very time consuming [16, 20, 21]. The purpose of this study was to implement a fast 3D gagCEST sequence at 7 T with a clinically feasible scan time and to evaluate the stability, reproducibility and clinical applicability of this method in articular cartilage in the knee.

## Methods

### Numerical simulations

The 3D gagCEST sequence implemented in this work is a pseudo-steady-state pulsed 3D gradient echo CEST sequence recently developed in our group [22]. The sequence was optimised through the Bloch-McConnell simulations [23]. The following sequence parameters were investigated: the number of saturation pulses, transmit field (B_1_^+^) amplitude and duty cycle. All other sequence parameters were fixed to the values that were eventually used for data acquisition [22]. Gradient and RF spoiling was simulated by setting the transverse magnetisation components to zero. Two-pool (free water and GAG) Bloch-McConnell equations were solved numerically [24] assuming the parameters in Table 1. GAG effect size was quantified by the pool difference method:

1 $$ \mathrm{GAG}=\mathrm{S}\ \left(0.9\ \mathrm{ppm},{\mathrm{M}}_{\mathrm{A}}=0\right)-\mathrm{S}\ \left(0.9\ \mathrm{ppm},{\mathrm{M}}_{\mathrm{A}}=1\right) $$

**Table 1.** Overview of parameters for Bloch-McConnell equation simulations

	Water	GAG
T_1_ (s)	1.2	1*
T_2_ (ms)	40	10
∆w (ppm)	0	0.9
M_0_ (%)	-	0.27
R (Hz)	-	1000

*Fixed in simulation

where S(∆ω,M_A_) is the simulated signal in the z-spectrum at ∆ω = 0.9 ppm, and M_A_ is the simulated amplitude of the GAG compartment. The saturation parameters were chosen to achieve an optimal GAG effect size, but with as low as possible acquisition time and within the limitations of the RF amplifier duty cycle.

### MRI data acquisition

Eight healthy volunteers without a history of knee pain or trauma and five patients undergoing arthroscopy for repair of a focal knee cartilage defect were included in this study (approved by the medical ethics committee). Patients were selected within our specialised knee clinic of the University Medical Centre Utrecht. Patients undergoing an arthroscopy for cartilage repair on the femoral condyle were included for a pre-operative MRI. Exclusion criteria were as follows: history of cartilage repair, history of cruciate ligament tears or repair and/or trochlear/patellar cartilage damage. Informed consent was acquired from all the subjects after explaining the study procedures. MRI experiments were carried out on a 7.0-T whole-body scanner (Achieva; Philips Healthcare, Best, The Netherlands), using an in-house developed and built volume transmit coil and a dedicated 32-channel receiver wrap-around knee coil (MR Coils BV, Zaltbommel, The Netherlands).

The 3D gagCEST sequence included a pre-saturation module consisting of a train (*n* = 20) of sinc-shaped pulses (B_1_ = 2 μT, pulse length = 25 ms, duty cycle = 70%, based on simulations). The readout parameters were as follows: five-shot turbo field echo (TFE), TFE factor of 370, SENSE factor of 2, TR/TE/FA = 2.75 ms/1.4 ms/5 degrees, field of view = 140 × 150 × 135 mm^3^, resolution = 1 × 1 × 3 mm^3^, inter-shot T1 recovery time = 2 s, k-space centre-weighted acquisition, two dummy scans, and total acquisition time = 6 min 59 s.

The gagCEST images were acquired at 17 saturation offsets ranging from -900 Hz to 900 Hz (-3 ppm to 3 ppm), i.e. -900, -600, -425, -350, -275, -200, -75, -25, 0, 25, 75, 200, 275, 350, 425, 600 and 900 Hz. In addition, gagCEST images were acquired at offsets of -100 kHz and +100 kHz to normalise the CEST spectrum. The expected resonance frequency of the hydroxyl side groups of GAG is 0.9 ppm, which is 270 Hz at 7 T [14].

Signal stability tests were carried out in five healthy volunteers (mean age: 26 years, age range: 21 to 35 years, two males and three females). Each subject was scanned twice and during each session 19 gagCEST images were acquired at a single saturation offset of 0.9 ppm (270 Hz). Nineteen acquisitions were chosen to represent the same scan duration as for the gagCEST experiment with 19 different offsets.

The reproducibility of the measurement of the GAG effect was assessed in eight healthy volunteers (mean age: 24 years, age range: 21 to 30 years, three males and five females). Each subject was scanned twice within the same scan session.

The clinical applicability of the method was demonstrated by comparing the GAG effect size in healthy cartilage versus damaged cartilage in five patients before cartilage repair (age range: 21 to 41 years, all male, no significant/obvious varus or valgus leg axis). These patients were scanned up to 24 h prior to surgery. During surgery, cartilage defects were graded with the International Cartilage Repair Society (ICRS) grading scale (grade 0 to 4, 0 = no damage; 4 = full thickness cartilage defect) [25]. The ICRS grade was graded in the femoral cartilage because we solely included patients with defects in the cartilage of the femoral condyles. The cartilage on the healthy condyle was graded with ICRS grade 0.

### Image analysis

Data analysis was performed in MATLAB (R2016b, the MathWorks, Natick, MA, USA) with in-house developed processing scripts. The signal stability measurements were normalised with respect to the signal intensity of the first measurement. The signal stability was quantified from the averaged signal over all pixels in each of the three regions of interest (ROI): the medial condyle, the trochlear groove and the lateral condyle. These regions were also used for the quantification of the reproducibility of the GAG effect.

CEST spectra were B_0_ corrected using WASSR [26] and were normalised using the high off-resonant gagCEST images at ± 100 kHz. The B_0_ corrected and normalised spectra were fitted pixel-wise using a sum of three Lorentzians to account for the GAG, water and magnetisation transfer pools [27]. The GAG effect is expected to be around 270 Hz, for which we chose three offsets to represent that point (200, 275, 350 Hz). The amplitude of the GAG pool is averaged over these three offsets to avoid outliers in the fit.

A 3D segmentation of the cartilage was used to evaluate the GAG effect in the patient group. Both weight-bearing condyles were divided into four regions (medial/lateral/superior/inferior) and the regions where a defect was present, according to the surgeon’s notes, were used for the analyses. These defect regions also include the defect rim, which is of great interest for treatment planning. These defect regions were compared with the same regions on the healthy contralateral condyle. A detailed explanation of the analysis workflow and the described regions of interest are shown in the Appendix.

### Statistical analysis

Stability of the signal (i.e. the value of the CEST spectrum at 275 Hz, where the GAG effect is expected) is expressed with the coefficient of variation (CV), which was calculated by dividing the standard deviation by the mean of the signal. The coefficient of variation was calculated in the three aforementioned ROIs and was calculated for both acquired stability assessments. The reproducibility of the GAG effect size was assessed by means of Bland-Altman plots and correlation plots with corresponding intraclass correlation coefficients (ICC), i.e. the degree of absolute agreement among measurements (criterion-referenced reliability). To evaluate differences between healthy cartilage and damaged cartilage in the patients, a Wilcoxon signed rank test was applied.

## Results

### Simulation data

**Fig. 1.**
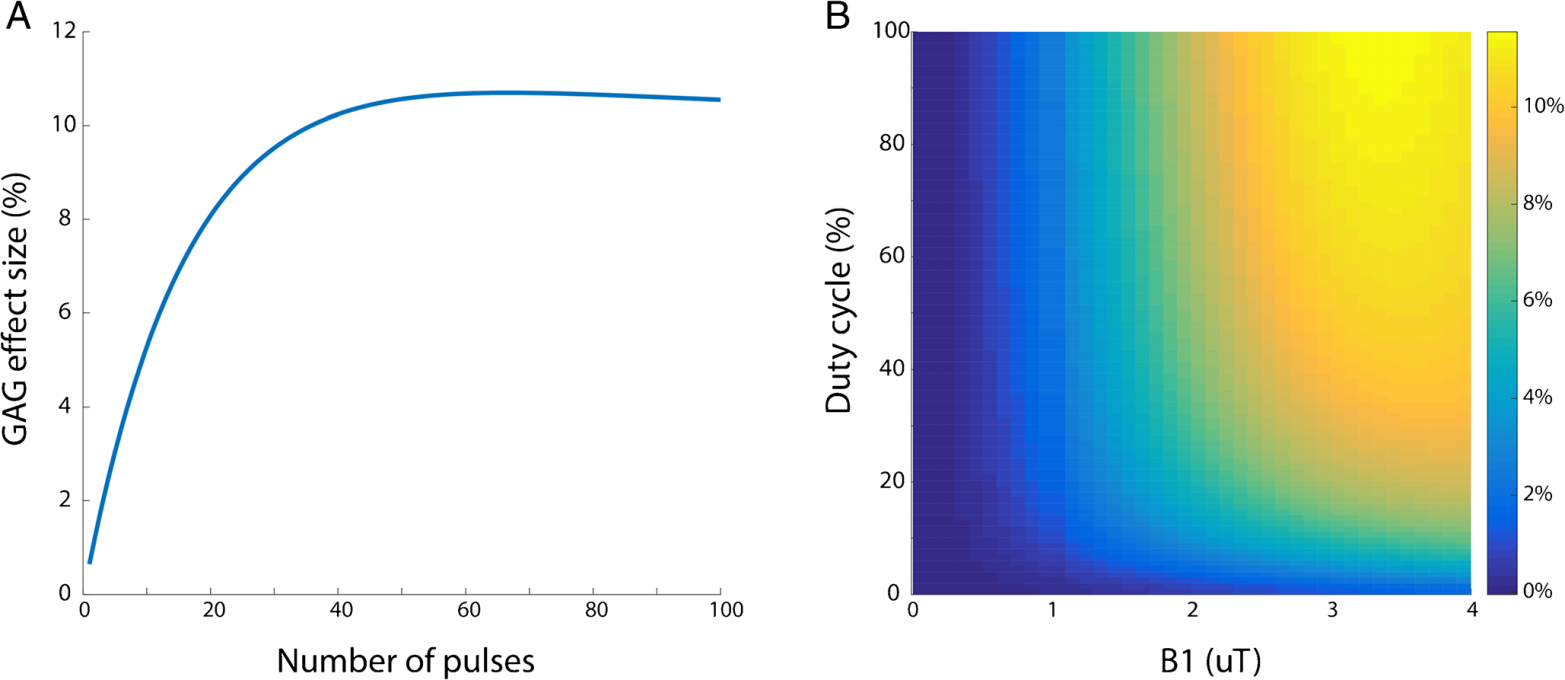
**(A)** The simulated GAG effect size (%) as a function of the number of pulses in the CEST pre-pulse. **(B)** The simulated 3D plot of GAG effect size (%) as a function of the RF duty cycle (of the CEST pre-pulse) and B_1+_ field amplitude

Figure 1 shows results of Bloch-McConnell simulations for the applied sequence. Twenty pulses were chosen as the number in the train, close to the maximum effect size for GAG but still within a clinically feasible acquisition time. A duty cycle of 70% was used to stay within RF amplifier duty cycle limits; 2 μT was chosen to approach the optimal effect size within the desired acquisition time. The combination of both leads to a maximum effect size of roughly 8 percent, which was in line with the chosen number of pulses in the pre-pulse train.

### Stability and reproducibility

The coefficients of variation of the signal stability assessments are reported in Table 2. The average CV in the medial condyle was 2.00%, the average CV in the lateral condyle was 2.25%, and the average CV in the trochlea was 1.40%.

**Table 2.** Stability assessments of GAG effect at 0.9 ppm in healthy volunteers

Subject	Age	Gender	Scan	Medial CV (%)	Trochlea CV (%)	Lateral CV (%)
1	21	F	1	1.61	0.88	1.23
			2	1.67	2.07	1.11
2	29	M	1	0.89	0.52	2.89
			2	1.25	0.52	0.73
3	35	M	1	3.2	1.96	1.74
			2	5.44	2.96	3.34
4	21	F	1	1.64	1.55	6.57
			2	1.54	0.57	0.88
5	25	F	1	1.08	1.54	1.49
			2	1.67	1.38	3.54
Mean coefficient of variation:				2.00	1.40	2.25

**Fig. 2.**
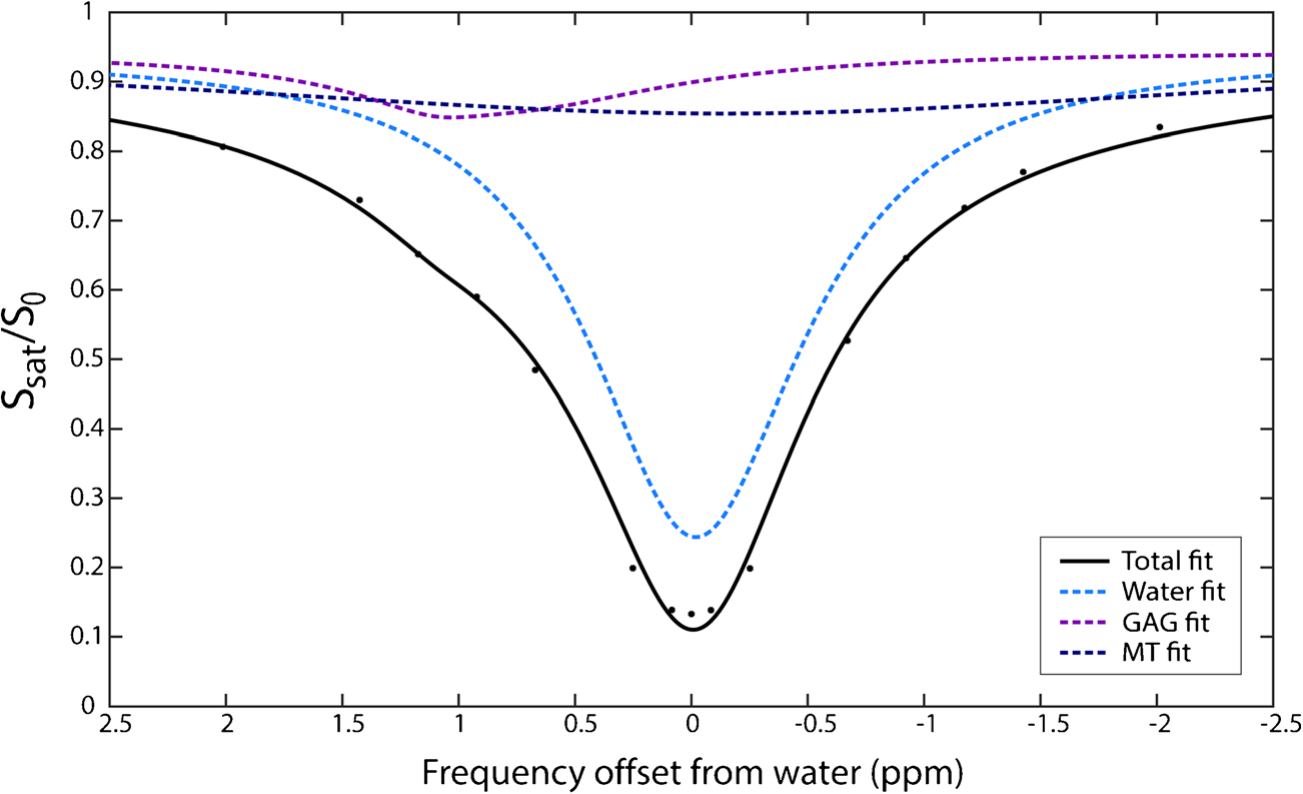
An example of the CEST spectrum and its three-pool Lorentzian decomposition. The *black line* shows the multi-Lorentzian fit of the three pools; acquired data are represented with *black dots*

Figure 2 shows an example of a fitted CEST spectrum, with the GAG, water and MT pools visualised in purple, light blue and dark blue, respectively. The GAG effect can be observed at the expected offset around 0.9 ppm.

**Fig. 3.**
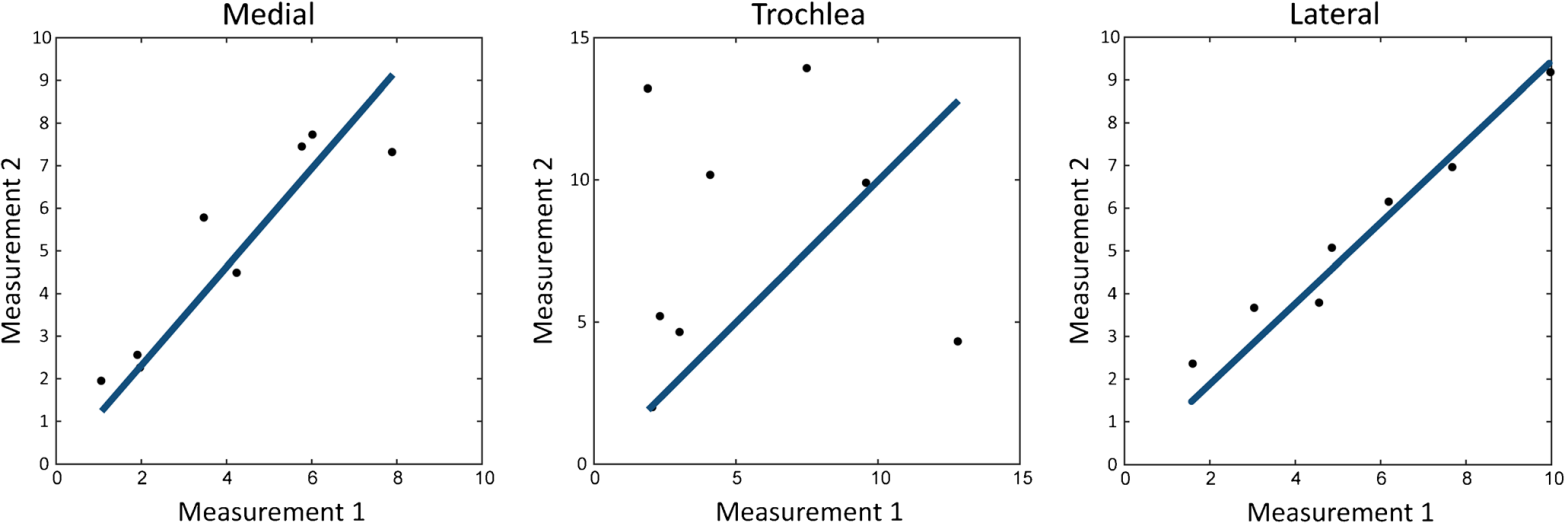
The correlation graphs of three assessed locations (medial condyle, lateral condyle and trochlear groove). ICC medial condyle: 0.87 (*p* = 0.0049), ICC trochlear groove: 0.063 (*p* = 0.43) and ICC lateral condyle: 0.97 (*p* < 0.001). Measurement 1 refers to the amplitude of the GAG fit in the first measurement; measurement 2 refers to the amplitude of the GAG fit in the second measurement

**Fig. 4.**
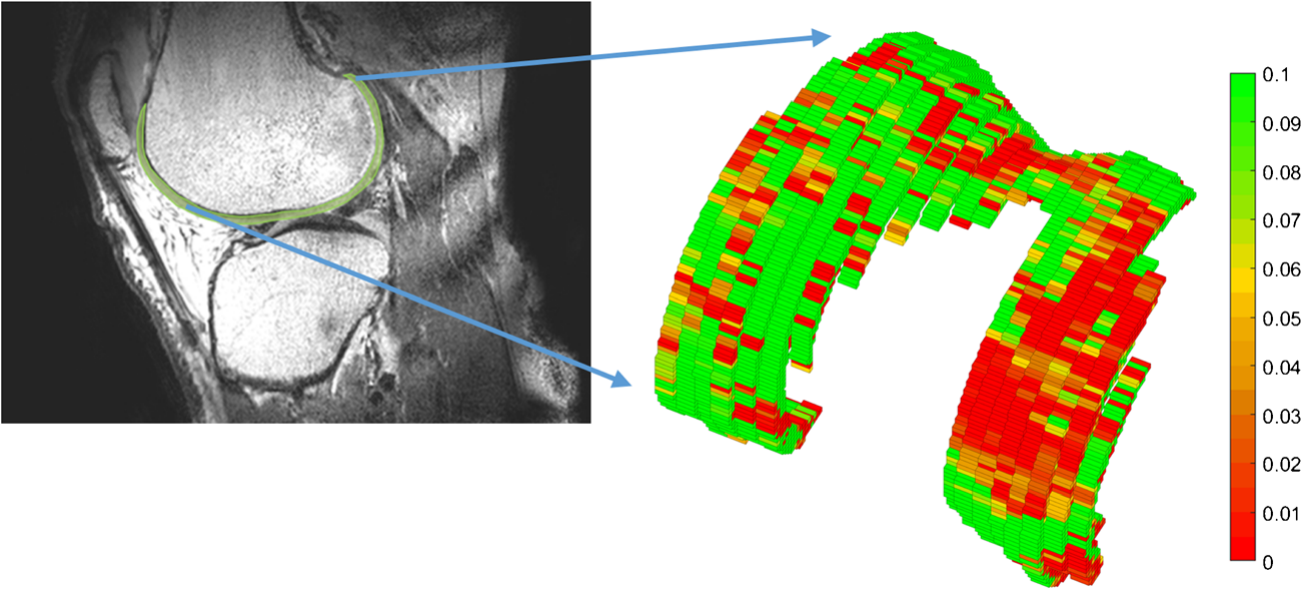
Bland-Altman plots of GAG effects in the medial and lateral condyle

The correlation plots in Fig. 3 show strong reproducibility in the lateral condyle (ICC = 0.97, *p* < 0.01) and the medial condyle (ICC = 0.87, *p* < 0.01). The ICC for the trochlear groove was weak (0.064, *p* = 0.43). Bland-Altman plots of the medial condyle and the lateral condyle are shown in Fig. 4. Bland-Altman analysis was not carried out in the trochlear groove because of the poor ICC. The Bland-Altman analyses show that there is no proportional bias between the two measurements.

### Clinical applicability

**Fig. 5.**
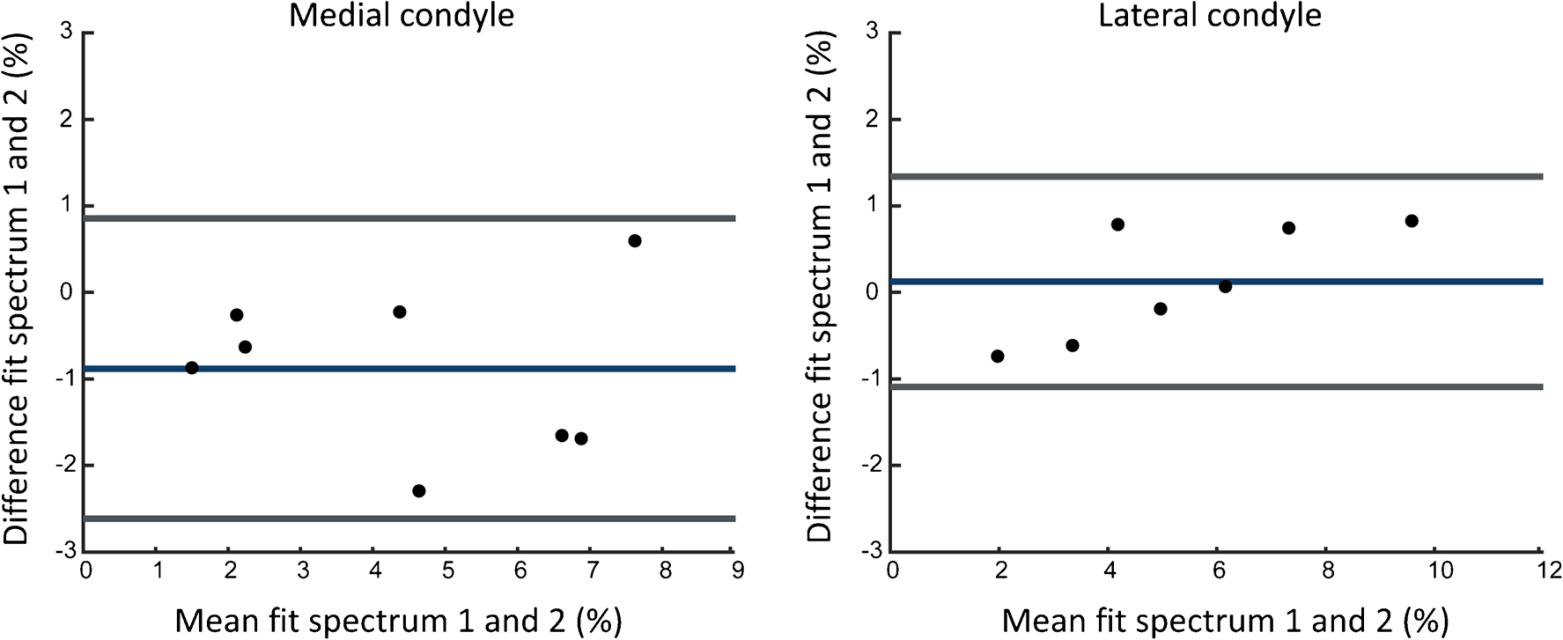
The 3D segmented GAG map of articular cartilage in the knee of a patient with an ICRS grade IV defect on the medial side of the knee

**Fig. 6.**
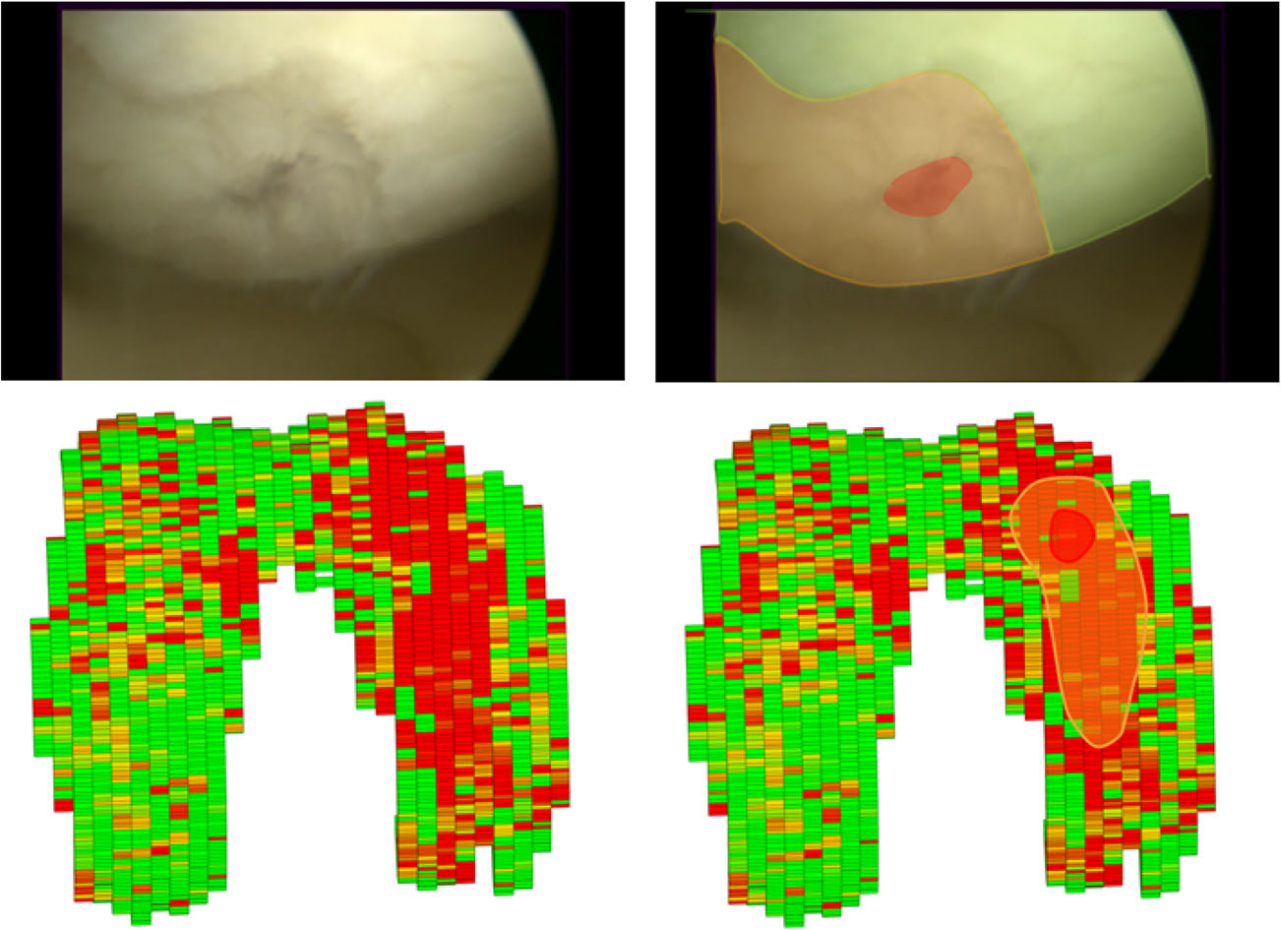
Comparison of the arthroscopic view and gagCEST map. *Left upper corner* shows the arthroscopic view of the knee of patient 1. The defect (*red*) and corresponding defect rim (*orange*) are highlighted in the image on the *upper right*. The lower left shows the gagCEST map of this patient, where the defect is clearly visualised. The same regions are highlighted again, with the defect in *red* and the defect rim in *orange*

A 3D segmented model of the knee cartilage of a patient is shown in Fig. 5. A difference in GAG effect in this patient was observed between the medial and the lateral sides. This specific patient had an ICRS grade IV defect in the medial condyle, which corresponds with the gagCEST findings. An artroscopic view of this patient and the corresponding 3D gagCEST map is shown in Fig. 6. The ICRS grades and GAG effects of all patients are summarised in Table 3. The ICRS grade ranged from 3 to 4 (> 50% thickness defects to full thickness cartilage defects). The GAG effect of healthy cartilage ranged from 2.6% to 12.4% and the GAG effect of damaged cartilage ranged from 1.3 to 5.1%. The GAG effect in damaged cartilage was significantly different (*p* < 0.05) from that in healthy cartilage.

**Table 3.** Comparison of GAG effect in cartilage repair patients: comparison of cartilage on damaged side of the knee versus cartilage on the healthy side of the knee. Difference between groups is statistically significant (*p* < 0.05)

No.	Age (years)	BMI (kg/m^2^)	ICRS grade	Defect location	Defect size (cm^2^)	Defect origin	GAG effect healthy condyle	GAG effect defective condyle
1	38	21.1	4	MFC	3	No trauma, gradual increase of pain	12.0 (5.7 – 21.2)	5.1 (0.1 – 11.8)
2	21	22.5	4	LFC	2	Distortion trauma	12.4 (5.0 – 21.6)	1.3 (0 – 7.5)
3	25	23.0	3	LFC	1.5	Cartilage damage after removal of meniscal lesion	9.3 (2.2 – 20.1)	1.8 (0 – 8.8)
4	41	29.5	4	MFC	4	Distortion trauma	2.6 (0 – 11)	2.5 (0 – 9.7)
5	26	22.9	4	LFC	1.5	Rotational trauma	3.7 (0.2 – 10.8)	1.4 (0 – 7.3)

MFC = medial femoral condyle; LFC = lateral femoral condyle; GAG effect is expressed as a median and interquartile range

## Discussion

This study presents a fast 3D gagCEST sequence with full cartilage coverage, which can quantify the GAG effect in healthy volunteers and patients. The data were acquired within seven minutes and shown to be stable and reproducible. Moreover, the method could differentiate healthy from damaged cartilage in patients before their cartilage repair surgery.

The main goal of this study was to present a fast 3D gagCEST sequence. The acquisition time for the gagCEST sequence used in this study was 6 min 59 s, because a pseudo-steady state sequence was applied with an optimised number of saturation pulses. Other 3D sequences were published with scan times ranging from 11 min [20] to almost 15 min [16]. The latest study of the group of Trattnig reported a scan time of 19 min, albeit with better resolution compared with our study [21]. Note that a higher resolution reduces the signal to noise and is more prone to artefacts related to motion of the knee. All sequences published used the same or a comparable number of offsets and comparable field of view. We chose to implement an in-plane resolution of 1 × 1 mm^2^ to minimise partial volume effects in the directions with the most curvature of the cartilage. This came with the drawback that the slice thickness needed to be 3 mm to achieve a sufficient SNR. Several other 3D gagCEST studies also implemented a comparable slice thickness of 3 mm [15, 16] or 5 mm [28]. An isotropic voxel size would be more ideal for 3D visualisation purposes, but this can only be achieved with a lower in-plane resolution or with much longer scan times.

This sequence was optimised using Bloch-McConnell simulations. Our goal was to minimise the scan time, which could lead to a sub-optimal CEST effect size. The number of pulses in the pre-pulse train could be increased to 60 for optimal effect size, as shown in Fig. 1A. However, this would increase the shot time to 5.4 s, which increases the acquisition time per offset by 8 s. This increase of 20% in effect size (8% to 10%) would lead to a 40% increase in total scan time (6:59 to 9:54). In our study we did not increase the scan time and selected a B_1+_ field amplitude of 2 μT and DC of 70% to obtain the maximum achievable effect size of 8%.

We applied a Lorentzian fitting algorithm for quantification of the GAG effect. We chose Lorentzian fitting to achieve better discrimination between the water peak and metabolite peak, in this case GAG. Because we expect GAG to resonate at 0.9 ppm [14], which is only 270 Hz upfield from the water peak, Lorentzian fitting was chosen. Lorentzian fitting also decreases the influence of B_0_ inhomogeneities [29]. Previous literature used MTR asymmetry as a method for quantification, which is prone to these B_0_ inhomogeneities [15, 16]. We used WASSR to correctly centre all CEST spectra as recommended by previous gagCEST studies [30].

The reproducibility in the lateral and medial femoral condyles was very good, which is promising for implementation in clinical practice. However, one should notice the poor reproducibility in the trochlear groove. The reproducibility in the trochlea was much lower compared to the condyles, which was also shown at 7 T in a study from Schreiner and colleagues [21]. The area around the trochlear groove is prone to movement of the patella. We speculate that this movement could be the cause of the poor reproducibility of the CEST spectra and their respective fits. Larger muscles could lead to more muscle twitches, ultimately leading to movement of the structures attached to the muscle, in this case the patella. The poor reproducibility could possibly be explained by this phenomenon. In addition, a 3-mm slice thickness could lead to volume averaging with surrounding tissue, especially in tissue with a high curvature such as the trochlea. Another limitation of this study is that measurements were only done on severe defects (ICRS grade III or IV) and healthy cartilage (ICRS grade 0). Because of the small population and the inclusion criteria for cartilage repair surgery in this study, no other defects were observed and gagCEST values of mild cartilage defects (ICRS grade I-II) are absent.

The GAG effect value varied across the included healthy volunteers and patients. The range of GAG effect values is rather large in patients, healthy cartilage ranging from 2.6% to 12.4%, compared with 1.3% to 5.1% for damaged cartilage. A similar range is observed in healthy volunteers (1.6% to 13.9%), which raises the question whether every volunteer had completely healthy cartilage. These ranges could indicate that there are underlying factors that affect the GAG effect, for instance age, gender or BMI, as has been suggested in other studies [31, 32]. Due to possible confounding effects of these factors, we chose not to compare the gagCEST values of patients with healthy volunteers.

Detection of the range of GAG effect values could be an interesting tool for osteoarthritis research, for monitoring of disease but also for earlier diagnosis. Therefore, a next step in this research would be an analysis of the GAG effect in patients with cartilage defects, ranging from small focal defects to osteoarthritic knees. This will reveal the value of gagCEST sequences in clinical practice and the patient characteristics affecting the GAG effect. In conclusion, this study presents a fast gagCEST sequence that is stable and reproducible and shows clinical value.

## Electronic supplementary material

ESM 1 (DOCX 65 kb)

## Abbreviations

CV: Coefficient of variation
GAG: Glycosaminoglycans
gagCEST: Glycosaminoglycan chemical exchange saturation transfer
ICC: Intraclass correlation coefficient
ICRS: International Cartilage Repair Society
OA: Osteoarthritis
