# Supplementary material for: Detection of early cartilage damage: feasibility and potential of gagCEST imaging at 7T
Source: Eur Radiol. 2018 Jan 30;28(7):2874–81. doi: 10.1007/s00330-017-5277-y (PMC5986839; doi:10.1007/s00330-017-5277-y)
Supplement: Supplementary file 1 — (DOCX 65 kb) [file 330_2017_5277_MOESM1_ESM.docx]

**Supplementary material**

**Appendix: assessment of defect locations**

We chose to assess the defect location and the contralateral healthy location in a standardized way, to
make sure we are not cherry-picking the results. In order to do this, we divide the condyle into four regions, as noted below. We select the quadrants in which the defect is located for the damaged side and select the same quadrants on the contralateral healthy side for comparison.

A: defect located on medial condyle

B: division of both condyles in four parts: superior-lateral, superior-medial, inferior-lateral

and inferior-medial

C: defect is located in in two parts of the condyle: inferior-lateral and inferior-medial (visualized in

orange). Same parts are used on contralateral healthy condyle to assess differences (visualized in green).
